# Hydrogen Sulfide Attenuates Carbon Tetrachloride-Induced Hepatotoxicity, Liver Cirrhosis and Portal Hypertension in Rats

**DOI:** 10.1371/journal.pone.0025943

**Published:** 2011-10-14

**Authors:** Gang Tan, Shangha Pan, Jie Li, Xuesong Dong, Kai Kang, Mingyan Zhao, Xian Jiang, Jagat R. Kanwar, Haiquan Qiao, Hongchi Jiang, Xueying Sun

**Affiliations:** 1 Department of General Surgery, The Hepatosplenic Surgery Center, The First Affiliated Hospital of Harbin Medical University, Harbin, China; 2 Department of ICU, The First Affiliated Hospital of Harbin Medical University, Harbin, China; 3 Department of Hepatobiliary Surgery, Affiliated Qianfoshan Hospital of Shandong University, Jinan, China; 4 Laboratory of Immunology and Molecular Biomedical Research, Centre for Biotechnology and Interdisciplinary Biosciences, Institute for Technology and Research Innovation, Deakin University, Geelong, Victoria, Australia; 5 Department of Molecular Medicine and Pathology, Faculty of Medical and Health Sciences, The University of Auckland, Auckland, New Zealand; Singapore Institute for Clinical Sciences, Singapore

## Abstract

**Background:**

Hydrogen sulfide (H_2_S) displays vasodilative, anti-oxidative, anti-inflammatory and cytoprotective activities. Impaired production of H_2_S contributes to the increased intrahepatic resistance in cirrhotic livers. The study aimed to investigate the roles of H_2_S in carbon tetrachloride (CCl_4_)-induced hepatotoxicity, cirrhosis and portal hypertension.

**Methods and Findings:**

Sodium hydrosulfide (NaHS), a donor of H_2_S, and DL-propargylglycine (PAG), an irreversible inhibitor of cystathionine γ-lyase (CSE), were applied to the rats to investigate the effects of H_2_S on CCl_4_-induced acute hepatotoxicity, cirrhosis and portal hypertension by measuring serum levels of H_2_S, hepatic H_2_S producing activity and CSE expression, liver function, activity of cytochrome P450 (CYP) 2E1, oxidative and inflammatory parameters, liver fibrosis and portal pressure. CCl_4_ significantly reduced serum levels of H_2_S, hepatic H_2_S production and CSE expression. NaHS attenuated CCl_4_-induced acute hepatotoxicity by supplementing exogenous H_2_S, which displayed anti-oxidative activities and inhibited the CYP2E1 activity. NaHS protected liver function, attenuated liver fibrosis, inhibited inflammation, and reduced the portal pressure, evidenced by the alterations of serum alanine aminotransferase (ALT), aspartate aminotransferase (AST), hyaluronic acid (HA), albumin, tumor necrosis factor (TNF)-α, interleukin (IL)-1β, IL-6 and soluble intercellular adhesion molecule (ICAM)-1, liver histology, hepatic hydroxyproline content and α-smooth muscle actin (SMA) expression. PAG showed opposing effects to NaHS on most of the above parameters.

**Conclusions:**

Exogenous H_2_S attenuates CCl_4_-induced hepatotoxicity, liver cirrhosis and portal hypertension by its multiple functions including anti-oxidation, anti-inflammation, cytoprotection and anti-fibrosis, indicating that targeting H_2_S may present a promising approach, particularly for its prophylactic effects, against liver cirrhosis and portal hypertension.

## Introduction

Hydrogen sulfide (H_2_S) has displayed many physiological and pathological activities [1]. Administration of H_2_S limited myocardial infarct size caused by ischemia reperfusion injury (IRI) [2], [3], suppressed development of gastric ulcer [4], alleviated hypoxic pulmonary hypertension [5], attenuated neuronal injury [6], and prevented the development of hypertension [7]. An H_2_S-releasing molecule, GYY4137, protected endotoxic shock by decreasing the production of proinflammatory cytokines including tumor necrosis factor-α (TNF-α) and interleukin-6 (IL-6) [8]. The multiple actions of H_2_S mainly involve inhibiting oxidative stress [9], production of lipid peroxidation and inflammatory factors [4], and activating ATP sensitive potassium (K_ATP_) channels [10].

H_2_S has also shown regulatory effects on hepatic physiology and pathology [11]. H_2_S is endogenously produced in mammalian tissues by cystathionine γ-lyase (CSE) and cystathionine β-synthase (CBS) [1], but CBS accounts for only 3% of H_2_S production in livers [12]. CSE-derived H_2_S contributes to hepatic arterial buffer response, mediates vasorelaxation of the hepatic artery via activation of K_ATP_ channels [13], and modulates biliary bicarbonate excretion [14]. H_2_S has exhibited anti-inflammatory and cytoprotective activities against hepatic IRI [15], and protected acetaminophen-induced hepatotoxicity in mice [16]. Diallyl trisulfide, an H_2_S-releasing chemical, protected from carbon tetrachloride (CCl_4_)-induced liver injury [17], [18].

The reduction of H_2_S production and CSE expression is related to the development of increased intrahepatic resistance and portal hypertension in a rat model of liver cirrhosis induced by CCl_4_ [19], and H_2_S counteracts the impaired vasodilation and hepatic stellate cell contraction, which contribute to the dynamic component of portal hypertension [20]. These two studies have focused on the regulatory effects of H_2_S in already established portal hypertension mainly by its vasodilative activities via activating K_ATP_ channels. As mentioned in one report [20], liver fibrosis represents the main causative factor in portal hypertension in cirrhosis, but the role of H_2_S in the development of cirrhosis remains unclear. CCl_4_ has been widely used to induce liver injury and cirrhosis in animal models, as it is rapidly metabolized into the trichloromethyl radical by cytochrome P450 (CYP) 2E1, and the reactive intermediate attacks membrane lipids, resulting in the formation of lipid peroxide molecules and necrosis of hepatocytes [21]. Given that H_2_S displays anti-oxidative, anti-inflammatory and cytoprotective activities, an anti-fibrotic effect against pulmonary fibrosis [22], and a regulatory effect on hypoxic pulmonary hypertension [23] and portal hypertension [19], [20], we hypothesized that H_2_S might have a protective effect against CCl_4_-induced acute hepatotoxicity, and the resulting liver cirrhosis and portal hypertension.

## Methods

### Ethics Statement

All the procedures and care administered to the animals have been approved by the institutional ethic committee, under a permit of animal use (SYXK20020009) in the First Affiliated Hospital of Harbin Medical University, compliance with the Experimental Animal Regulations by the National Science and Technology Commission, China.

### Animals

Male Wistar rats (weighing 210-230 g) were supplied by the Animal Research Center at the First Affiliated Hospital of Harbin Medical University, Harbin, China. They were housed in the animal facility with a 12-h-light/-dark cycle and the temperature was maintained at 22-23°C.

### Acute hepatotoxicity experiment

The rats received phenobarbital sodium (0.35 g/L) in drinking water for 3 days, followed by a single i.p. injection of CCl_4_ (Nanjing Chem. Ltd., Nanjing, China) diluted in equal volume of paraffin oil at a dose of 3 ml/kg body weight. Then the rats were randomly assigned to 3 groups (each group had 6 rats): control, NaHS and PAG, receiving an i.p. injection of 1 ml of physiological saline, NaHS (Sigma-Aldrich) solution (14 µmol/kg body weight), or PAG (Sigma-Aldrich) solution (50 mg/kg body weight), respectively. The injection was repeated every 6 h. The rats were killed 48 h after CCl_4_ administration. Six untreated rats served as healthy controls. Blood was collected via cardiac puncture, and serum was prepared by centrifugation at 2000 × g for 10 min, and stored at -80°C. Livers were divided into two parts, one fixed in 10% buffered formalin, the other stored at -80°C.

### Induction of liver cirrhosis

Liver cirrhosis was induced by CCl_4_ as described previously [20], [21]. Briefly, rats received phenobarbital sodium (0.35 g/L) in drinking water for 3 days, followed by i.p. injections of 1 ml/kg body weight of CCl_4_ diluted in an equal volume of paraffin oil twice a week for 12 weeks.

### Prevention experiment

Thirty-six rats receiving i.p. injections of CCl_4_ as above were randomly assigned into 3 groups (each had 12 rats): saline, NaHS and PAG, receiving i.p. injections of 1 ml of saline, NaHS solution (10 µmol/kg body weight) and PAG solution (30 mg/kg body weight), respectively, every two days for 12 weeks, starting on the same day of CCl_4_ administration. 300 µl of blood samples were collected by tail tipping at indicated time points. At completion of the experiments, the rats were weighed, and the portal pressure measured as below, and 300 µl of blood sample was collected from the portal vein. The spleen was weighed as % of bodyweight and used as a parameter for portal hypertension. Blood was collected via cardiac puncture, and then the liver and spleen were collected. Serum and liver samples were prepared and stored as above.

### Measurement of portal pressure

The methodology has been described previously [24]. Briefly, under anesthesia by an i.p. injection of sodium pentobarbital (50 mg/kg body weight), rats underwent laparotomy, and a PE-tube (23G, 0.6×30 mm) was inserted over the ileocolic vein and advanced toward the confluence of the portal and splenic veins. This cannula was used to monitor portal pressure for 5 min by a Medlab-Ug4Cs bio-signal processing system (Nanjing Medease Science and Technology co., Ltd, Nanjing, China).

### Treatment experiment

Rats with liver cirrhosis induced by 12-week administration of CCl_4_ as above were randomly assigned to 3 groups (each had 6 rats): saline, NaHS and PAG, receiving daily injection of 1 ml of saline, NaHS solution (10 µmol/kg) and PAG solution (30 mg/kg), respectively, for 5 days. At completion of the experiments, the portal pressure measured, and then serum and liver samples were prepared.

### Measuring H_2_S production in livers and H_2_S concentration in sera

The methods have been described previously [15], [25]. All samples were assayed in triplicate and H2S was calculated against a calibration curve of NaHS (0.122–250 µmol/L). The H2S producing activity was expressed as µmole of H2S formed/g tissues.

### RT-PCR analysis

The analysis of CES mRNA by RT-PCR has been described previously [15]. A pair of primers 5′- GAC CTC AAT AGT CGG CTT CGT TTC -3′ and 5′- CAG TTC TGC GTA TGC TCC GTA ATG -3′ to generate a 618 bp product of CSE mRNA. A 497 bp PCR product was amplified from glyceraldehyde 3-phosphate dehydrogenase (GAPDH) cDNA as an internal control, with a pair of primers: 5′-GAAGGTGAAGGTCGGAGT-3′ and 3′-TGAAACCATAGCACCTTCC-5′. PCR products underwent a 2% agarose gel electrophoresis, and the density of each band was evaluated using the gel image analyzer (UV ChemiDOC, USA). The relative density of mRNA levels was calculated as the following formula: band density/ GAPDH band density.

### Measurement of serum parameters

The serum levels of alanine aminotransferase (ALT), aspartate aminotransferase (AST), hyaluronic acid (HA) and albumin were measured with an auto-biochemical analyzer (Toshiba, Japan). The serum levels of TNF-α, IL-6, interleukin-1β (IL-1β) and soluble intercellular adhesion molecule-1 (ICAM-1) were measured with ELISA kits (BPB Biomedicals, Inc., USA).

### Measurement of CYP2E1 activity in livers

The CYP2E1 activity was assayed using *p*-nitrophenol (Sigma-Aldrich) as a substrate as described previously [26].

### Measurement of hepatic malondialdehyde (MDA) and glutathione (GSH)

Liver tissue was homogenized in a buffer containing 9 volumes of 0.15 mol/L KCl-1.0 mmol/L EDTA to obtain 1∶10 (w/v) homogenates. Homogenates were then centrifuged at 10,000 g (4°C) for 30 min to collect the supernatant for determining the concentrations of MDA, GSH and total protein. MDA was evaluated by the thiobarbituric acid reactive substances (TBARS) method [27]. The final concentration of MDA was expressed as µmol/g protein. GSH were measured using commercially available kits (Jiancheng Institute of Biotechnology, Nanjing, China) and expressed as nmol/mg protein. Protein concentration of liver homogenate was determined by the Bradford method, using bovine serum albumin as a standard [28].

### Measurement of hepatic hydroxyproline

Hepatic hydroxyproline was measured as described previously [29].

### Histological analysis

Formalin-fixed liver specimens were embedded in paraffin, sectioned, stained with hematoxylin and eosin (HE) or Masson, and examined under light microscopy. Quantifying liver fibrosis was performed by measuring blue pixels of the images taken from Masson-stained sections. Ten photographs (200 × magnification) were randomly taken from each liver at fixed exposure time and conditions. The pictures were saved as JPEG (Joint photographic experts group). The histogram function in Adobe Photoshop CS4 was used to count the blue pixels, and the numbers of red pixels were recorded for each picture.

### Western Blot analysis

The methodology has been described previously [15]. The Abs against CSE or α-smooth muscle actin (α-SMA) (Santa Cruz Biotechnology, Inc. Santa Cruz, CA) were used. The levels of proteins were normalized with respect to band density of β-actin, as an internal control.

### Statistics

Results were expressed as mean values ± standard deviation (SD). A one-way analysis of variance (ANOVA) followed by the post-hoc Dunnett's test was used for evaluating statistical significance (SPSS 17.0). A value of P<0.05 was considered significant.

## Results

### H_2_S protects livers from CCl_4_-induced acute toxicity

Administration of NaHS significantly attenuated, while PAG further raised, the elevated serum levels of ALT and AST by CCl_4_ (Table 1). The impaired liver function by CCl_4_ and the protective effects of H2S were supported by the histological alterations (Figure S1). The liver sections from healthy control rats showed normal histology (Figure S1A). CCl_4_-treated livers had focal midzonal necrosis and ballooning degeneration (Figure S1B). NaHS attenuated the necrosis and vacuolization (Figure S1C), while PAG further aggravated the hepatotoxicity and caused more inflammatory cells infiltration (Figure S1D). NaHS also significantly reduced the activity of CYP2E1, the major enzyme to metabolize CCl_4_, in livers from CCl_4_-treated rats, compared with saline, while either CCl_4_ or PAG had no effects on its activity (Table 1). CCl_4_-treated rats had a significant higher level of hepatic MDA (a marker of lipid peroxidation) and a significantly lower level of hepatic GSH, compared to healthy controls. NaHS significantly attenuated the increase of hepatic MDA, and restored hepatic GSH, but PAG only had slight effects on hepatic MDA and GSH, in CCl_4_-treated rats, compared with saline (Table 1).

**Table 1:** Serum ALT and AST, and hepatic CYP2E1, MDA and GSH in hepatotoxicity experiment*.

	Healthy control	CCl_4_		
		saline	NaHS	PAG
	(n = 6)	(n = 6)	(n = 6)	(n = 6)
Serum ALT (IU/L)	36.2±7.5	245.8±36.5^a^	149.6±25.7a,b	268.7±46.9a,b
Serum AST (IU/L)	78.9±11.4	657.0±96.1^a^	427.6±102.3a,b	835.2±133.5a,b
Liver CYP2E1(µmol/g protein)	2.8±0.2	2.7±0.4	1.4±0.3a,b	3.1±1.0
Liver MDA (µmol/g protein)	1.6±0.3	5.9±1.2^a^	3.8±0.7a,b	6.3±1.5^a^
Liver GSH (µmol/g protein)	11.5±2.4	5.7±1.8^a^	10.7±3.0a,b	4.6±1.1^a^

*The blood and liver samples were collected from the rats 48 h after CCl_4_ administration. Data are expressed as means ± SD, and statistical analysis was performed by one-way ANOVA analysis followed by the post-hoc Dunnett's test.

a Significant difference (P<0.05) from healthy controls.

b Significant difference (P<0.05) from saline -treated rats.

### Serum levels of H_2_S, production of H_2_S and CSE expression in the acute experiment

**Figure 1:**
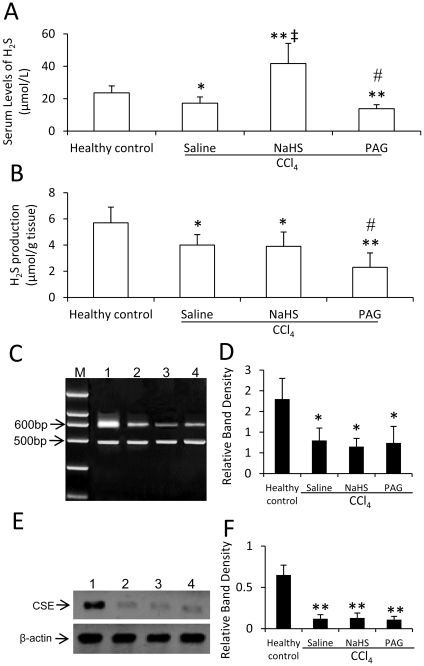
Serum levels of H_2_S, H_2_S production and CSE expression in CCl_4_-induced acute hepatotoxicity. Healthy rats, or CCl_4_-treated rats receiving administrations of saline, NaHS or PAG, were killed 48 h after CCl_4_ administration, blood and liver samples were collected. The levels of H_2_S in sera (A) and H_2_S producing activity in livers (B) were measured. (C-F) The expression of CSE mRNA (C, D) and protein (E, F) was detected in liver tissues from healthy rats (lane 1), or CCl_4_-treaed rats receiving administration of saline (lane 2), NaHS (lane 3) or PAG (lane 4), by RT-PCR (C, D) or Western blot analysis (E, F), respectively. M, DNA marker. (D) The density of each band from (C) was measured and compared to that of the internal control, GAPDH. (F) The density of each band from (E) was measured and compared to that of the internal control, β-actin. Results are expressed as mean ± SD (n = 6). Compared to the healthy controls, a significant difference is denoted by “*” , and a highly significant difference by “**” (P<0.001). Compared to rats treated with CCl_4_ + saline, a significant increase is denoted by “‡”, and a significant reduction is denoted by “#”.

The serum levels of H_2_S in CCl_4_ + saline-treated rats were significantly lower than that in healthy controls (P<0.05) (Fig. 1A), supported by that CCl_4_ significantly (P <0.05) reduced hepatic H_2_S producing activity (Fig. 1B). Hepatic expressions of CSE mRNA (Fig. 1C and D) and protein (Fig. 1E and F) were significantly (Both P<0.05) lower in CCl_4_-treated rats than that in healthy controls. NaHS significantly (P<0.001) increased the levels of H_2_S (Fig. 1A), but had no effect on hepatic H_2_S producing activity (Fig. 1B) and the expression of CSE mRNA (Fig. 1C and D) and protein (Fig. 1E and F), in CCl_4_-treated rats, compared saline. However, PAG significantly (Both P<0.05) reduced the levels of H_2_S (Fig. 1A) and H_2_S producing activity (Fig. 1B), but had no effect on the expression of CSE mRNA (Fig. 1C and D) and protein (Fig. 1E and F), in CCl_4_-treated rats, compared with saline.

### H_2_S production in cirrhotic rats induced by CCl_4_

**Figure 2:**
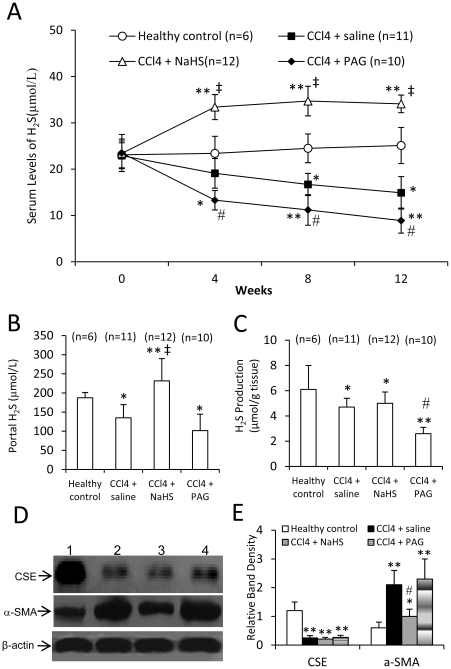
Serum H_2_S levels, H_2_S production, and expression of CSE and α-SMA in the prevention experiment. The rats were treated with CCl_4_ + saline, CCl_4_ + NaHS or CCl_4_ + PAG for 12 weeks. Untreated rats served as healthy controls. (A) Blood samples were collected at indicated time points, and the serum levels of H_2_S were measured. The statistical comparison between two groups was done at the respective time point. (B-E) The rats were killed at the completion of experiment. Blood samples were collected from portal vein, and livers harvested. The levels of H_2_S in sera from the portal vein (B), and H_2_S producing activity in livers (C) were measured. (D) The expression of CSE and α-SMA was detected in livers from healthy controls (lane 1), or rats treated saline+CCl_4_ (lane 2), NaHS+CCl_4_ (lane 3) or PAG+CCl_4_ (lane 4) by Western blot analysis. (E) The density of each band was measured and compared to that of the internal control, β-actin. Results are expressed as mean ± SD. n, number of samples. Compared to the healthy controls, a significant difference is denoted by “*”, a highly significant difference by “**” (P<0.001). Compared to saline + CCl_4_-treated rats, a significant increase is denoted by “‡”, and a significant reduction is denoted by “#”.

Among the rats in the prevention experiment, one from the CCl_4_ + saline group and two from the CCl_4_ + PAG group died before completion of the experiment, and were excluded from the study. Blood samples were collected at indicated time points to measure the serum levels of H_2_S in rats. The healthy rats had a stable serum level of H_2_S, while the H_2_S levels in CCl_4_-induced cirrhotic rats receiving injection of saline declined at week 4 after CCl_4_ administration, and continued dropping as the H_2_S levels were even lower at week 8 and 12 than that at week 4 (Fig. 2A). However, NaHS significantly (P<0.05) elevated the serum level of H_2_S at week 4 after CCl_4_ administration in CCl_4_-induced cirrhotic rats, compared with saline, and serum H_2_S was maintained at a higher level at week 8 and 12 (Fig. 2A). Administration of PAG resulted in significantly (All P<0.05) lower serum levels of H_2_S in CCl_4_-induced cirrhotic rats, than saline, at all the three time points (Fig. 2A). The portal serum levels of H_2_S at the completion of experiment was significantly (P<0.05) lower in CCl_4_-induced cirrhotic rats receiving injection of saline than the healthy controls (Fig. 2B). However, NaHS significantly (P<0.05) elevated, but PAG did not significantly (P>0.05) decrease, the portal serum levels of H_2_S, in CCl_4_-induced cirrhotic rats, compared with saline (Fig. 2B). We further examined the H_2_S producing activity (Fig. 2 C) in livers from CCl_4_-induced cirrhotic rat receiving injections of saline, NaHS or PAG, and the similar results were obtained as shown in the acute hepatotoxicity experiment (Fig. 1B), supported by the results of hepatic CES expression (Fig. 2D and E), where CCl_4_ significantly downregulated, while NaHS or PAG had no effect on, the expression of CES.

### H_2_S attenuates the impaired liver function and elevated inflammatory factors in cirrhotic rats

Cirrhotic rats induced by 12-week administration of CCl_4_ had significantly higher levels of ALT, AST, HA and lower level of albumin, than healthy controls (Table 2). However, simultaneous administration of NaHS resulted in significantly lower levels of ALT, AST and HA, and higher level of albumin, while PAG led to significantly higher levels of AST and HA, slight increase of ALT level and slight reduction of albumin level, in CCl_4_-induced cirrhotic rats, compared with saline (Table 2).

**Table 2:** Serum biochemicals and inflammatory factors in cirrhotic rats*.

	Healthy control	CCl_4_ + saline	CCl_4_ + NaHS	CCl_4_ + PAG
	(n = 6)	(n = 11)	(n = 12)	(n = 10)
ALT (IU/L)	34.4±8.9	198.8±26.7^a^	137.6±20.3a,b	208.5±36.4^a^
AST(IU/L)	80.3±12.5	466.7±46.4^a^	317.5±78.2a,b	515.0±97.6a,b
HA (µg/L)	113.5±20.1	482.6±51.4^a^	296.3±32.1a,b	549.0±47.3a,b
Albumin (g/L)	472.5±43.8	314±51.6^a^	443.7±80.5a,b	294.3±32.8^a^
TNF-α (ng/L)	43.9±6.8	143.5±21.4^a^	109.3±22.6a,b	176.3±28.5a,b
IL-1β(ng/L)	20.3±5.0	75.4±10.2^a^	50.6±9.8a,b	80.2±17.4^a^
IL-6 (ng/L)	87.0±15.9	297.1±55.4^a^	216.4±42.1a,b	320.6±51.4^a^
ICAM-1(ng/L)	69.2±8.7	180.8±19.7^a^	153.6±22.8a,b	207.2±31.3a,b

*Data are expressed as means ± SD, and statistical analysis was performed by one-way ANOVA analysis followed by the post-hoc Dunnett's test.

a Significant difference (P<0.05) from healthy controls.

b Significant difference (P<0.05) from CCl_4_+saline-treated rats.

Cirrhotic rats had significantly higher levels of proinflammatory cytokines including TNF-α, IL-1β and IL-6, and soluble ICAM-1 (Table 2), which have been shown to play important roles in development of cirrhosis. However, simultaneous administration of NaHS resulted in significantly lower levels of all the four parameters in CCl_4_-induced cirrhotic rats than saline, while PAG led to significantly higher levels of TNF-α and soluble ICAM, and slight increase of IL-1β and IL-6, in CCl_4_-induced cirrhotic rats, compared with saline (Table 2).

### H_2_S attenuates CCl_4_-induced liver cirrhosis and portal hypertension

**Figure 3:**
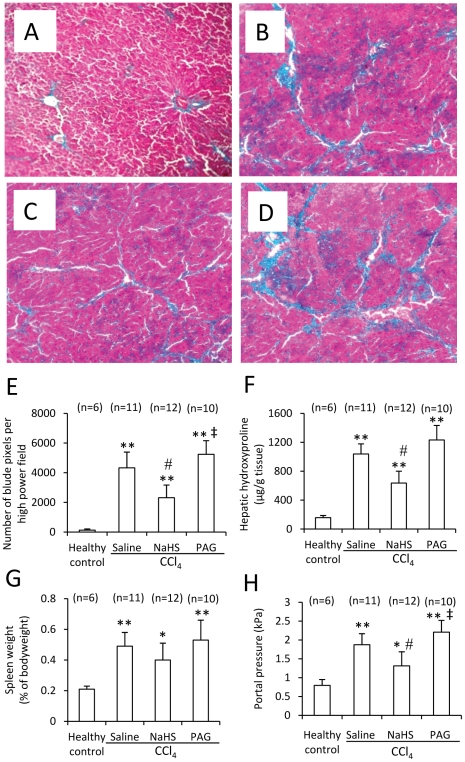
Histology of liver cirrhosis, hepatic hydroxyproline, spleen weight and portal pressure in the prevention experiment. Representative illustrations (200 × magnification) of Masson-stained liver sections were taken from healthy controls (A), or rats treated with CCl_4_ + saline (B), CCl_4_ + NaHS (C) or CCl_4_ + PAG (D), for 12 weeks. (E) The numbers of blue pixels of each image of the above Masson-stained liver sections were counted, and the average number of blue pixels for each liver was calculated. (F) The level of hydroxyproline in livers taken from the above rats was measured. (G) Each spleen was weighed as % of bodyweight. (H) The portal pressure of each was measured. Data were expressed mean ± SD. n, number of samples. Compared to the healthy controls, a significant difference is denoted by “*”, and a highly significant difference, by “**” (P<0.001). Compared to saline + CCl_4_-treated rats, a significant increase is denoted by “‡”, and a significant reduction is denoted by “#”.

The Masson-stained liver sections from healthy controls had almost no collagen fibers of blue color (Fig. 3A), but those from CCl_4_-induced cirrhotic rats injected with saline had abundant and widespread fibers of blue color (Fig. 3B). However, the liver sections from CCl_4_-induced cirrhotic rats injected with NaHS had fewer fibers (Fig. 3C), while those from PAG-injected cirrhotic rats (Fig. 3D) had even more fibers, compared with those from saline-injected cirrhotic rats. The numbers of blue pixels in Masson-stained liver sections were measured to quantify collagen fibers. CCl_4_ highly significantly (P<0.001) increased the number of blue pixels in liver sections (Fig. 3E). However, administration of NaHS resulted in a significant (P<0.05) lower number of blue pixels, but PAG, a significant (P<0.05) higher number of blue pixels, in CCl_4_-induced cirrhotic rats, compared with saline (Fig. 3E). We further measured hydroxyproline, a marker of fibrosis, and found that CCl_4_-treated rats had significantly (P<0.001) higher hepatic hydroxyproline content than the healthy controls. However, administration of NaHS significantly (P<0.05) reduced, but PAG did not significantly (P>0.05) increase, the hepatic hydroxyproline contents in CCl_4_-induced cirrhotic rats, compared with saline (Fig. 3F). Next, we detected hepatic expression of α-SMA, another marker of liver fibrosis, and found that the CCl_4_-treated rats had significantly higher hepatic expression of α-SMA, compared to the healthy controls. However, administration of NaHS significantly (P<0.05) downregulated, but PAG did not significantly (P>0.05) increase, hepatic expression of α-SMA in CCl_4_-induced cirrhotic rats, compared with saline (Fig. 2D and E).

The spleen weight and portal pressure were used as parameters for portal hypertension. The spleen weight of CCl_4_–treated rats was highly significantly (P<0.001) higher than that of healthy controls (Fig. 3G). However, administration of neither NaHS nor PAG resulted in significant difference in spleen weight of CCl_4_-induced cirrhotic rats, compared with saline (Fig. 3G). CCl_4_-induced cirrhotic rats had a significantly (P<0.001) higher portal pressure than the healthy controls (Fig. 3H). However, administration of NaHS resulted in a significant (P<0.05) lower level of portal pressure, while PAG, a significant (P<0.05) higher level of portal pressure, in CCl_4_-induced cirrhotic rats, than saline (Fig. 3H).

### The therapeutic effect of H_2_S on liver cirrhosis and portal hypertension

**Figure 4:**
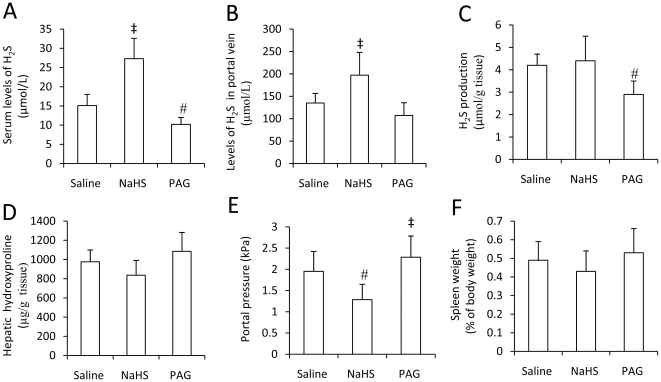
H_2_S levels and production, hepatic hydroxyproline, portal pressure and spleen weight in the treatment experiment. Liver cirrhosis was induced in rats as in Figure 3, and then the rats were assigned to three groups (Each group had 6 rats), and received daily injection of saline, NaHS or PAG, respectively, for 5 days, and then killed. (A) Blood samples were collected and the serum levels of H_2_S were measured. (B) Blood samples were collected from portal vein to measure the levels of H_2_S in portal vein (B). The hepatic H_2_S producing activity (C) and hydroxyproline contents (D) were measured. The portal pressure was measured (E), and each spleen was weighed as % of bodyweight (F). Results are expressed as mean ± SD. A significant increase from saline-treated rats is denoted by “‡”, and a significant reduction by “#”.

Given that H_2_S displayed a preventive effect against CCl_4_-induced liver cirrhosis and portal hypertension, we next investigated whether H_2_S could have a similar therapeutic effect in already established liver cirrhosis. Liver cirrhosis was induced in rats by 12-week administration of CCl_4_ as above, and then saline, NaHS or PAG was administered to the cirrhotic rats, respectively, for 5 days. Administration of NaHS significantly (Both P<0.05) elevated the levels of H_2_S in sera from the peripheral blood (Fig. 4A) and portal vein (Fig. 4B), but again had no effect on H_2_S production activity in livers (Fig. 4C). PAG significantly (P <0.05) reduced the levels of H_2_S in sera from the systemic circulation (Fig. 4A), marginally significantly (P = 0.07) reduced the levels of H_2_S in the portal vein (Fig. 4B), and significantly (P <0.05) reduced the H_2_S production in livers (Fig. 4C). NaHS or PAG had no significantly influence on the histological alterations of livers (Data not shown), and the hepatic hydroxyproline in NaHS or PAG-treated rats were not significant different from the saline-injected rats (Fig. 4D). However, administration of NaHS significantly (P<0.05) reduced the portal pressure, while PAG significantly (P<0.05) elevated the portal pressure, compared with saline (Fig. 4E), but their effects on spleen weight were not significant, compared with saline (Fig. 4F).

## Discussion

CCl_4_ has been widely used as a chemical model to induce hepatotoxicity, liver cirrhosis and portal hypertension, since it is metabolized into trichloromethyl radical, leading to increased lipid peroxidation, depletion of GSH, impaired hepatic anti-oxidant activity and necrosis of hepatocytes [21], [30]. The present study has demonstrated that CCl_4_ downregulated the expression of CSE, the major enzyme accounting for 97% of H_2_S production in livers [1], [12], [19], resulting in decreased hepatic H_2_S producing activity. Administration of NaHS, a donor of H_2_S, attenuated CCl_4_-induced acute hepatotoxicity, evidenced by the reduction of serum levels of AST and ALT, and attenuation of histopathological alterations, in accordance with the previous reports on its cytoprotective effects against myocardial IRI [2], endotoxin-induced cardiac injury [31], hepatic IRI [15], acetaminophen-induced hepatotoxicity [16], or gastric mucosa damage caused by stress [4], nonsteroidal anti-inflammatory drugs [32] or oxidative stress [33]. The protective effects of H_2_S may rely on its anti-oxidative activity by reducing the production of lipid peroxides, as NaHS inhibited the activity of CYP2E1, one of the major enzymes metabolizing CCl_4_ [21]. The vasodilative activity of H_2_S may also contribute this protective activity, as H_2_S-induced vasorelaxation [34] can improve microcirculation of livers, which help livers to get rid of excessive lipid peroxides. In addition, though not investigated herein, the anti-apoptotic activity of H_2_S as demonstrated against hepatic IRI [15], may also contribute to this action.

The effects of CCl_4_ on H_2_S production were shown to be accumulative as the serum levels of H_2_S declined in a time-dependent course. NaHS had no effect on either hepatic CSE expression or H_2_S producing activity, and led to a relatively higher but stable level of H_2_S in sera, implying that the body has the ability to adjust endogenous production of H_2_S or get rid of excessive H_2_S, which has been recognized as a “toxic gas” in environmental pollution [35]. In both the acute and prevention experiments, PAG, an irreversible inhibitor of CSE, significantly suppressed the H_2_S producing activity and reduced the H_2_S levels, thus showing opposite effects to NaHS on liver function, lipid peroxides, and liver fibrosis, but had no impact on CSE expression, in accordance with previous reports [3], [15].

The present study has for the first time demonstrated the anti-fibrotic activity of H_2_S against liver cirrhosis, evidenced by reduced number of collagenous fibers in livers, and hepatic hydroxyproline content and expression of α-SMA. Besides the direct mechanism on fibrogenesis, the protective effect of H_2_S on hepatic injury also contributes to its anti-fibrotic activity. In addition, NaHS inhibited the production of inflammatory factors including TNF-α, IL-1β, IL-6, soluble ICAM-1. The anti-inflammatory effect, particular inhibition of IL-6 production by H_2_S, can contribute to its anti-fibrotic activity, as IL-6 is upregulated in cirrhotic livers and contributes to the fibrogenic process in an autocrine/paracrine manner [36].

The regulation of sinusoidal resistance depends on the contraction of hepatic stellate cells (HSCs) around sinusoidal endothelial cells [19]. It has been demonstrated that H_2_S is an autocrine mediator involved in regulating HSCs contraction and maintaining portal venous pressure by targeting KATP channels [19]. H_2_S can counteract the impaired vasodilation and HSC contraction, thus reducing portal hypertension, cirrhotic livers [20]. The results presented herein further support the effects of H_2_S on portal hypertension as administration of NaHS showed prophylactic and therapeutic effects in reducing portal hypertension. The protective effects of H_2_S on liver cirrhosis may also attributes to this effect in the prevention experiment, as liver fibrosis represents the main causative factor in portal hypertension [20]. However, H_2_S did not show significant therapeutic effects on liver cirrhosis in the treatment experiment, based on the histological alterations and hepatic hydroxyproline contents in cirrhotic rats, indicating H_2_S may not have the ability to reverse the process of liver fibrosis.

In conclusion, the present study has for the first time systemically investigated the potential protective role of H_2_S on CCl_4_-induced acute hepatotoxicity, the prophylactic effect of H_2_S on long-term CCl_4_-induced cirrhosis and portal hypertension, and the therapeutic effect of H_2_S on portal hypertension, by its multiple functions including anti-oxidation, anti-inflammation, cytoprotection and anti-fibrosis. The results indicate that targeting H_2_S may present a potent approach, particularly for its prophylactic effects, against liver cirrhosis and portal hypertension. Preclinical trials by applying some promising H_2_S-relesasing chemicals have already been launched though at their infancy. For instance, diallyl trisulfide, a stable H_2_S donor and organic polysulfide compound [37], has been shown to protect from carbon tetrachloride (CCl_4_)-induced liver injury [17], [18]. However, cautions must be taken as H_2_S has been recognized as a “toxic gas” [35], and more preclinical trials are required before H_2_S-releasing agents reach the clinics for use in preventing liver cirrhosis and portal hypertension.

## Supporting Information

Figure S1 Histology of hepatotoxicity induced by CCl4. Representative illustrations (200 × magnification) of HE-stained liver sections were taken from healthy Wistar rats (A), or CCl4-treaed rats receiving administration of saline (B), NaHS (C) or PAG (D).
(TIF)
