# Supplementary figures and images for: Hydrogen Sulfide Attenuates Carbon Tetrachloride-Induced Hepatotoxicity, Liver Cirrhosis and Portal Hypertension in Rats
Source: PLoS One. 2011 Oct 14;6(10):e25943. doi: 10.1371/journal.pone.0025943 (PMC3195078; doi:10.1371/journal.pone.0025943)

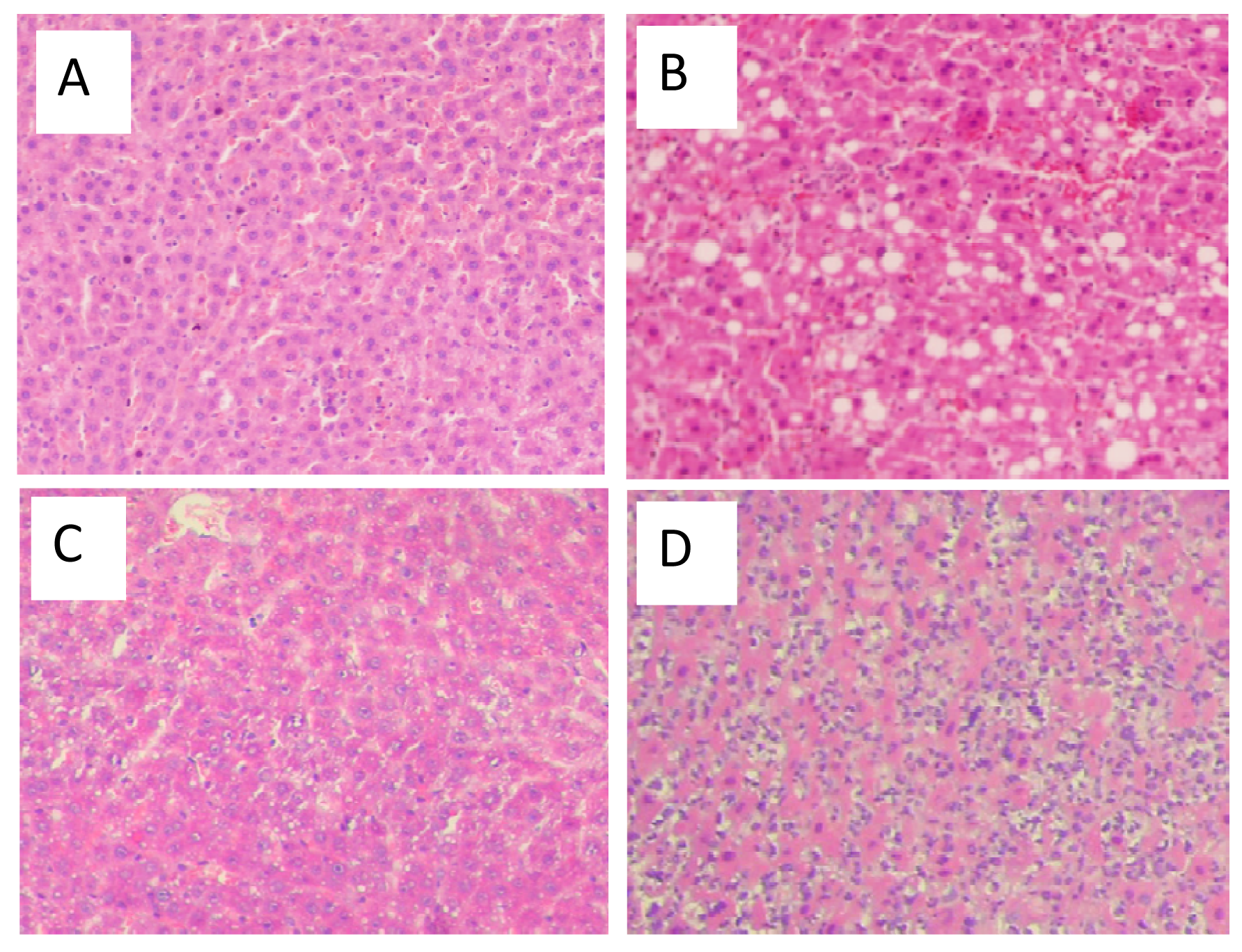

Supplement: Figure S1 — Histology of hepatotoxicity induced by CCl4. Representative illustrations (200 × magnification) of HE-stained liver sections were taken from healthy Wistar rats (A), or CCl4-treaed rats receiving administration of saline (B), NaHS (C) or PAG (D). (TIF) [file pone.0025943.s001.tif]
